# Detecting Lies via a Theme-Selection Strategy

**DOI:** 10.3389/fpsyg.2018.02775

**Published:** 2019-01-11

**Authors:** Nicola Palena, Letizia Caso, Aldert Vrij

**Affiliations:** ^1^Department of Human and Social Sciences, University of Bergamo, Bergamo, Italy; ^2^Department of Psychology, University of Portsmouth, Portsmouth, United Kingdom

**Keywords:** Theme-Selection strategy, within-subjects comparisons, lie detection, HUMINT interviewing, investigative interviewing, strategic interviewing

## Abstract

Most of deception research has focused on past events that were either completely truthful or a complete fabrication. However, people often tell a mixture of truths and lies. This could enable investigators to make within-subjects comparisons between different themes discussed in one interview, which we examined in the current experiment. Seventy-three participants took part in the experiment and were asked to either tell the truth about two themes, or to tell the truth about one theme and lie about the second theme in a HUMINT setting. Results showed that examining the differences in the amount of detail provided by the interviewees for each theme- obtained through a Theme-Selection strategy (a within-subjects measure)- yielded stronger results than examining differences between truth tellers and liars based on the entire interview without accounting for themes (between-subjects measure). The present study therefore highlighted the effectiveness of within-subjects measurements to both discriminate truth tellers from liars and to discover which section of a statement is false.

## Introduction

Research has shown that cues to deception are faint and unreliable (DePaulo et al., 2003) and that people’s ability to detect lies is low (Bond and DePaulo, 2006, 2008). Consequently, scholars switched their attention to the development of interviewing techniques aimed at enhancing the amount of information revealed by the interviewee and to elicit cues to deception (Vrij and Granhag, 2012; Vrij, 2014). Most of them are based on memory research and cognition and have shown potential in discriminating truth tellers and liars (Vrij, 2008, 2015, 2018; Ost et al., 2015; Rosenfeld, 2018).

One of the new developments is to focus on differences within an individual rather than between individuals. So-called within-subjects comparisons can reduce problems caused by interpersonal differences (Nahari and Pazuelo, 2015; Vrij, 2016) and are preferred by both practitioners and scholars (Nahari and Vrij, 2014, 2015; Nahari, 2016; Vrij, 2016). Comparisons within an individual can be made in different ways (for a detailed and recent review of within-subjects comparisons, see Vrij, 2016, 2018). An example is to compare specific variables within an interviewee statement. For example, research has shown that the proportion of verifiable details compared to non-verifiable details (Nahari et al., 2014) was higher for truth tellers than for liars. Similarly, truth tellers reported a higher proportion of complications (operationalized as complications/(complications + common knowledge details + self-handicapping strategies) than liars (Vrij et al., 2018).

Another example is the use of the reverse order technique, where the interviewee is firstly asked for a free recall and then to report the same event starting from the end and going back toward the beginning (Fisher and Geiselman, 1992; Fisher et al., 2014). Research has shown that truth tellers report more reminiscences than liars when asked to recall the event in reverse order (see Vrij, 2016).

We examined another within-subjects measure, described below, based on the finding that interviewees often tell a mixture of truths and lies (Maguire and John, 1995; Leins et al., 2013). That is, interviewees are honest in one section of their statement in which they describe one event (or topic) but lie in another section of the statement where they discuss a second event (or topic) (Palena et al., 2018). Discussing various topics in one interview is not uncommon in intelligence interviews (Deeb et al., 2017).

In a recent study introducing an intelligence type interview setting, truth tellers honestly reported two events, whereas liars lied about one event and told the truth about the other event (Deeb et al., 2017). All participants were interviewed twice, always with a free recall in the first interview, and with either a free recall or a set of specific questions in the second interview. It was found that liars’ accounts included less repetitions than truth tellers’ accounts for both events, particularly when the second interview was conducted via specific questioning.

The present study was similar to Deeb et al. (2017) in that our lying participants also told the truth about one event (theme 1, non-critical event) but lied about another event (theme 2, the critical event). However, our study differs from Deeb et al. (2017) in at least two ways. First, we only interviewed the participants once. Second, rather than focusing on consistency, we focused on the amount of information revealed by the interviewees, based on research showing that liars are typically less detailed and forthcoming than truth tellers (DePaulo et al., 2003; Vrij, 2008; Hartwig et al., 2014).

We postulate that differences between truth tellers and liars should become more evident when the interviewer examines differences between the specific themes (Theme-Selection Strategy) than when the interviewer considers the statement as a whole without accounting for specific themes. The differences in detail provided between the themes within each individual’s statement can then be used to decide whether someone is lying, and which part of the statement is the lie. As such, the comparison is not between truth tellers’ and liars’ entire statement, but between the interviewee’s answers regarding the different themes of the interview. It thus becomes a within-subjects comparison. We predict that truth tellers, who will tell the truth about both themes, will show no difference in the amount of information provided when talking about the two themes, whereas liars, who tell the truth about the non-critical theme but lie about the critical theme, will report more information when talking about the non-critical theme (truth) than when talking about the critical theme (lie) (Hypothesis 1). We further predict that comparing interviewees’ answers to the two themes (within-subjects measure) is more efficient for lie detection purposes than comparing truth tellers’ and liars’ answers taken as a whole (without accounting for themes, between-subjects measure) (Hypothesis 2). The reason for Hypothesis 2 is two-fold. First, within-subjects measures are typically more diagnostic than between-subjects measures (Vrij, 2016). Second, only the within-subjects measure is a true comparison between truths and lies, the between-subjects measure is a comparison between a total truth (truth tellers) and a mixture of truths and lies (liars).

## Materials and Methods

### Participants

All participants were university students. An announcement was made at the beginning of lectures and a list of names and email addresses was obtained. The students were informed that in case of a convincing performance during the interview they would be offered one additional credit for their exam (all participants received the credit). Seventy-three participants took part in the experiment (61 females and 12 males). Age of this sample ranged from 20 to 45 years, *M* = 22.06 (*SD* = 2.95), median = 22. However, data screening showed that five participants were outliers^1^ in terms of details reported. The new sample, on which all analyses were conducted, therefore consisted of 68 participants (56 females and 12 males). The age ranged from 20 to 25, *M* = 21.71 (*SD* = 0.99), median = 21.50.

### Design

We employed a 2 (Theme: non-critical vs. critical, within-subjects) by 2 (Veracity: truth telling vs. lie, between-subjects) mixed design. For the factor Theme, the participant had to report information about the structure and activities of the criminal organization (non-critical theme) and information about the hideout of the boss, his routines etc. (critical theme). For the factor Veracity, the participants either told the truth or lied about the critical theme (all participants told the truth about the non-critical theme). Additionally, since the amount information remembered (see below) can influence the amount of revealed information, the number of pieces of information for the two themes remembered by the participants were entered as covariates. The amount of revealed information was the dependent variable.

### Procedure

Participants were informed that they would participate in an experiment mirroring a HUMINT interview. Upon arrival, each participant was welcomed and asked to read and sign the consent form if s/he decided to participate. S/he was then told that s/he had to play the role of a secret agent whose agency was trying to dismantle a criminal organization. All participants were informed that there was a spy working against their agency, whose goal was to protect the criminal organization. However, justifications for the following interview differed between the conditions (as in Deeb et al., 2017).

Truth tellers were informed that the interviewer could be trusted. They were asked to report all information honestly so that the interviewer would be fully informed about the interviewee’s experiences and could conclude that the interviewee was not hiding anything. Liars were told that there was a risk that the interviewer was a spy which they needed to fool. Therefore, they were asked to adhere to the following instructions: To make an honest impression on the interviewer they had to report honestly everything relating to the structure of the criminal organization, its activities and components (non-critical section, 18 pieces of information, the truth). However, they were asked to lie about the boss’ hideout, activities and routines (critical event, 18 pieces of information, the lie).

The assignment to the Veracity condition was alternated, meaning that the first participant was assigned to the honest condition, the second to the lying condition, the third to the honest condition and so on.

The experimenter then gave each participant (both truth tellers and liars) a file containing all the information about the criminal organization that the agency possessed. The participant was asked to study it in detail and to remember it for the following interview. The participant was then left alone to study the file and was asked to inform the experimenter when s/he had memorized the file information.

After 15 min, the experimenter returned to the room and made sure that the participant understood the role and instructions. The participant was then asked to complete a memory-check questionnaire, where open ended questions regarding each of the 36 pieces of information were asked (*e.g., “At what time does the boss leaves his hideout?”*). After that, s/he was given 10 min to prepare for the interview. Then, the participant was informed that the interview would start and was reminded that s/he would receive the additional study credit only when performing well during the interview. Eventually, all participants received the credit. All interviews were video-recorded. When the interview was finished, the participant was told that the experiment was concluded, and s/he was asked to complete another memory-check. This contained two additional questions compared to the first memory-check. First, the participant was asked how motivated s/he was to convince the interview that s/he was telling the truth on a 7-point Likert scale ranging from one (not at all) to seven (totally). Second, the participant was asked whether s/he believed that s/he appeared credible to the interviewer on a 7-point Likert scale ranging from one (not at all) to seven (totally). Eventually, the participant was thanked, debriefed about the aims of the experiment, and told that s/he received the additional study credit.

### Interview

Three people acted as interviewers, and each of them interviewed about one-third of the sample. All interviewers were blind to the study hypotheses and experimental conditions. The interviewers were trained and carried out simulated interviews before interviewing actual participants, similar to Oleszkiewicz et al. (2014). This to make sure that: (a) they followed the structure of the protocol; (b) would not improvise or make changes during the interview; (c) and kept a constant demeanor during the interviews (Mann et al., 2013). The interviewer started introducing herself, then asked the participant to briefly tell something about themselves, such as their hobbies and interest. This question was asked to put the interviewee at ease and to avoid that the first moment of the interview were influenced by the context (such as the presence of the camera). The interviewer then asked the following free recall question: *“As you know, our agency is investigating a criminal organization led by the Passatante clan. Tell me everything you know about this criminal organization in as much detail as possible.”* This was followed by the question: *“Is there anything you would like to add*?*”* The interviewer then asked two follow up questions to elaborate on the two themes. *“Ok, now tell me everything you know about the structure of the organization, such as its components, roles, and activities”* (non-critical theme) and *“Ok, now tell me everything you know about the Boss of the criminal organization”* (critical theme).

The order of the two follow-up questions was counterbalanced. Additionally, the second follow-up question was followed by another open-ended question: *“Is there anything you would like to add?.”* After this, the participant was thanked, and the interview ended.

### Coding

First, all video interviews were transcribed. Two experienced coders, both blind to the experimental conditions and the aims of the study, coded the first 22 (about 30%) interviews for the presence of information regarding the non-critical and the critical themes revealed by the interviewee throughout the interview. Each piece of information was counted only once. The coding took place using a checklist that included all the 36 pieces of information provided to the interviewee, similar to Oleszkiewicz et al. (2014). For both the non-critical and critical themes, the scores on this checklist could range from 0 to 18. The total score accounting for both themes together could thus range from 0 to 36. Yet, liars may opt for the strategy to be as detailed as possible to appear credible. Therefore, they could report pieces of information, not present in the story they were initially given. For this reason, the coders also counted the number of pieces of information not initially given.

We calculated inter-rater reliability on 30% of the transcripts^2^ using the two-way random, single measure, model: ICC (2,1) (Shrout and Fleiss, 1979; Landers, 2015). The absolute agreement for the non-critical theme was of ICC = 0.99, and for the critical theme, ICC = 0.93, showing high agreement. At this point, any disagreement was discussed by the two coders and resolved. Then, one coder coded the remaining 70% of the transcripts. Pieces of information were divided into “true” information and “false” information for manipulation checks (see below). However, for hypothesis testing, the information was separated for information concerning the non-critical and critical themes but not for veracity. One aim of the present study was to mirror a situation where the interviewers did not have previous knowledge that would give them the opportunity to detect any statement-evidence inconsistency.

## Results

### Manipulation Check

Participants reported high levels of motivation to perform well during the interview (*M* = 6.09, *SD* = 0.91) and thought that they appeared credible (*M* = 4.85, *SD* = 0.95). Motivation did not differ between truth tellers (*M* = 6.08, *SD* = 1.02) and liars (*M* = 6.09, *SD* = 0.78), *t*(64.47) = –0.048, *p* = 0.96, *d* = –0.01 [–0.49, 0.47]. Perceived credibility differed between truth tellers (*M* = 5.08, *SD* = 0.94) and liars (*M* = 4.59, *SD* = 0.91), *t*(66) = 2.179, *p* = 0.03, *d* = 0.53 [0.04, 1.01]. Additionally, liars revealed more pieces of information that were false (*M* = 11.44, *SD* = 4.14) than truth tellers (*M* = 1.05, *SD* = 1.09), *t*(34.845) = 13.758, *p* < 0.001, Cohen’s *d* = –3.53 [–4.24, –2.73]. This means that the Veracity manipulation was successful^3^.

Previous research found that interviewers can have an influence on the interviewees’ answers (Mann et al., 2013). A linear mixed model analysis was conducted. The mean intercept and the interviewer condition (3 levels: interviewer 1, vs. interviewer 2, interviewer 3, between-subjects) were the fixed factors. Intercepts were the random factor. The number of reported pieces of information was the dependent variable. The fixed effect of interviewer condition was not significant, *F*(2, 65) = 0.17, *p* = 0.84.

### Hypothesis Testing

**FIGURE 1:**
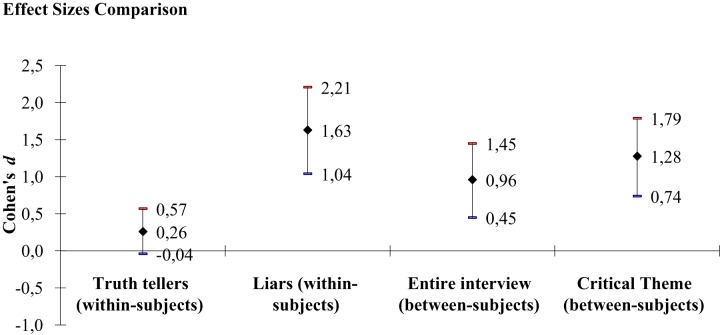
Comparison of between-subjects and within-subjects effect sizes, with 95% confidence intervals. Cohen’s *d* was computed with original metrics without controlling for the covariates.

A linear mixed-model was conducted with Theme (non-critical vs. critical, within-subjects) and Veracity (truth telling vs. lying, between-subjects) as fixed factors and the total amount of *reported* information (information included in the original story plus information added by the participants) throughout the interview as dependent variable. The amount of information for the non-critical and critical themes *remembered* by the interviewees before the beginning of the interview were the covariates. The intercepts were the random effect. Effect sizes are reported in Table 1 and Figure 1 for comparisons purposes.

**Table 1:** Between-subjects vs. within-subjects effect sizes and parameter estimates comparisons.

		95% C.I.			95% C. I.	
Between-subject effects	*Cohen’s d^a^*	LL	UL	*Parameter estimates^b^* (*SE*)	LL	UL
Veracity [both themes]	0.96	0.45	1.45	–0.83 (0.26)	–1.34	–0.32
Veracity [critical theme]	1.28	0.74	1.79	–1.65 (0.39)	–2.43	–0.87
**Within-subject effects**						
Theme [both veracity conditions]	0.86	0.54	1.17	–1.42 (0.21)	–1.83	–1.02
Theme [truth tellers]	0.26	–0.04	0.57	–0.55 (0.30)	–1.14	0.04
Theme [liars]	1.63	1.04	2.21	–2.29 (0.29)	–2.87	–1.71


*Effects were obtained from the linear mixed model used for hypothesis testing.*

*^a^Cohen’s d was computed with original metrics without controlling for the covariates. ^b^Parameter estimates were obtained from the linear mixed model used for hypothesis testing.*

The Theme main effect was significant, *F*(1, 62) = 47.11, *p* < 0.001. Participants reported more pieces of information when talking about the non-critical theme (*M* = 16.26, *SD* = 1.90) than when talking about the critical theme (*M* = 13.56, *SD* = 4.03) (Table 2).

**Table 2:** Means, standard deviations, and 95% confidence intervals for the factor “Theme” obtained from the linear mixed model used for hypothesis testing.

			95% C.I.				95% C.I.	
Theme	*M*	*SD*	LL	UL	Est. Marginal Mean	*SE*	LL	UL
Non-critical	16.26	1.90	15.80	16.72	16.14	0.33	15.48	16.80
Critical	13.56	4.03	12.58	14.53	13.29	0.33	12.63	13.95


*Means and SD were computed without adjusting for the covariates and for the factor “Veracity.” Estimated marginal means show the mean effect for the factor Theme, adjusting for the effect of the covariates and factor Veracity.*

The main effect for Veracity was also significant, *F*(1, 62) = 10.08, *p* = 0.002. Truth tellers reported more pieces of information overall (*M* = 31.86, *SD* = 4.26) than liars (*M* = 27.53, *SD* = 4.76) (Table 3).

**Table 3:** Means, standard deviations, and 95% confidence intervals for the factor “Veracity” obtained from the linear mixed model used for hypothesis testing.

			95% C.I.				95% C.I.	
	*M*	*SD*	LL	UL	Est. Marginal Mean	*SE*	LL	UL
Truth tellers	31.86	4.26	30.42	33.30	15.54	0.37	14.80	16.29
Liars	27.53	4.76	25.81	29.25	13.89	0.36	13.16	14.62


*Means and SD were computed without adjusting for the covariates and for the factor “Theme.” Estimated marginal means show the mean effect for the factor Veracity, adjusting for the effect of the covariates and factor Theme.*

**FIGURE 2:**
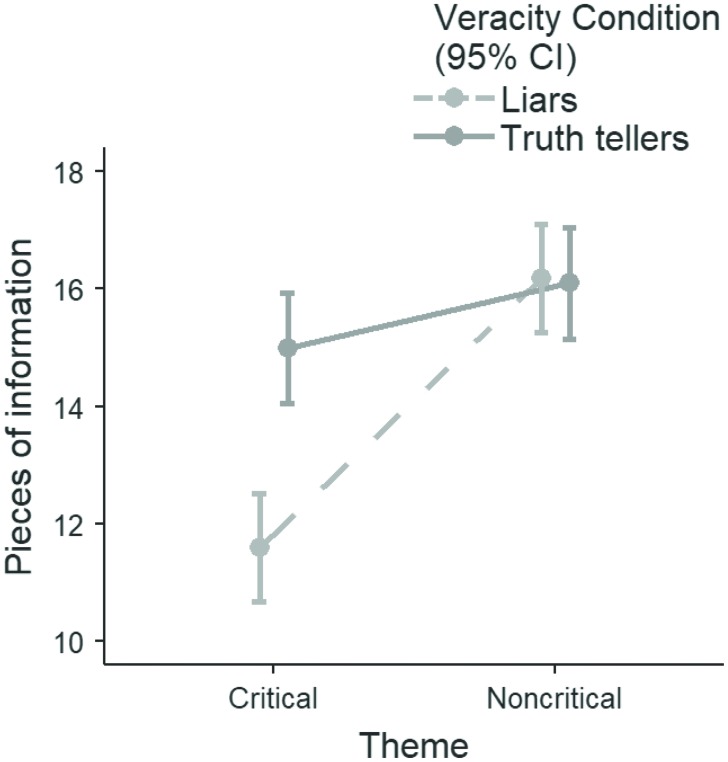
Theme by Veracity interaction. Y-axis shows the amount of revealed information.

The Theme by Veracity interaction was also significant, *F*(1, 62) = 17.63, *p* < 0.001 (Figure 2). Simple effect analyses (Table 4) showed that truth tellers reported a similar amount of information for the non-critical (*M* = 16.25, *SD* = 1.90) and critical (*M* = 15.61, *SD* = 2.87) themes, *F*(1, 62) = 3.47, *p* = 0.07, LogBF_(10)_ = –0.523^4^. In contrast, liars reported more pieces of information when talking about the non-critical theme (*M* = 16.28, *SD* = 1.92) than when talking about the critical theme (*M* = 11.25, *SD* = 3.93), *F*(1, 62) = 62.66, *p* < 0.001, LogBF_(10)_ = 12.70. These findings support Hypothesis 1.

**Table 4:** Means, standard deviations, and 95% confidence intervals of simple effects analyses for truth tellers and liars.

	Non-critical				Critical			
			95% C.I.				95% C.I.	
	*M*	*SD*	LL	UL	*M*	*SD*	LL	UL
Truth tellers	16.25	1.90	15.60	16.89	15.61	2.87	14.64	16.58
Liars	16.28	1.92	15.59	16.97	11.25	3.93	9.83	12.67


*All statistics are reported without adjusting for the covariates.*

We conducted the same analyses without entering the covariates into the model and obtained similar results. The Theme main effect, *F*(1, 66) = 52.92, *p* < 0.001, Veracity main effect, *F*(1, 66) = 15.64, *p* < 0.001, and Theme by Veracity interaction, *F*(1, 66) = 31.76, *p* < 0.001, were again all significant. Simple effect analyses were again not significant for truth tellers, *F*(1, 66) = 1.43, *p* = 0.23, but significant for liars, *F*(1, 66) = 78.70, *p* < 0.001.

We conducted the same analyses without entering the covariates into the model and without excluding outliers. We obtained similar results except that for the Veracity main effect. The Theme main effect, *F*(1, 71) = 25.97, *p* < 0.001, and the Theme by Veracity interaction, *F*(1, 71) = 11.54, *p* = 0.001, were again significant. Simple effect for truth tellers were again not significant, *F*(1, 71) = 1.46, *p* = 0.23, whereas simple effect for liars were again significant, *F*(1, 71) = 35.58, *p* < 0.001. The Veracity main effect, however, was no longer significant, *F*(1, 71) = 1.42, *p* = 0.23.

In Hypothesis 2 we predicted that the within-subjects measure would be more effective to discriminate truth tellers from liars than the between-subjects measure. An appropriate way to test this hypothesis is to compare the effect sizes of the two methods. The effect sizes are a measure of the magnitude of differences, where larger effect sizes imply larger differences [see for overviews about the importance of effect sizes and its comparison with significance testing, du Prel et al. (2009) and Fritz et al. (2012)]. Such an approach has already been used in previous research (Deeb et al., 2017).

Cohen (1988, 1992) states that an effect of *d* > 0.80 is large and noticeable by observers. For the between-subjects measure focused on the entire interview, we obtained a Cohen’s *d* = 0.96 [0.45, 1.45]. For the within-subjects measure, we obtained a Cohen’s *d* = 0.26 [–0.04, 0.57] for truth tellers and a Cohen’s *d* = 1.63 [1.04, 2.21] for liars (Table 1 and Figure 1).

It is also important to compare truth tellers and liars when focusing on the critical theme only, as this is the only theme about which the participants were asked to either lie or tell the truth.

An ANCOVA with Veracity (truth tellers vs. liars) as the factor, the amount of remembered information for the critical theme as the covariate, and the amount of revealed information for the critical theme as the dependent variable showed that the effect for Veracity was significant, *F*(1, 64) = 17.75, *p* < 0.001, *d* = 1.28 [0.74, 1.79]. Truth tellers reported more pieces of information than liars (Table 4).

Hypothesis 2 can be only partially supported for the following two reasons. First, although the effect size for the within-subjects measure, when looking at liars, was larger than the effect size obtained for the between-subjects measure focusing on the entire interview, both were large. Second, there is an overlap between the confidence intervals of the two effect sizes. However, if two confidence intervals overlap, there is still the possibility that a significant difference is present. In the present hypothesis, it was predicted that within-subjects measures are better than between-subjects measures. As we only found differences within liars’ responses (we did not find differences within truth tellers’ responses), this corresponds to the null hypothesis that the difference in the amount of reported information for the critical theme compared to the amount of reported information for the non-critical theme for liars, is not different from the difference between truth tellers and liars when analyzing the amount of reported information for the critical theme only. In short, (liars’ total details for the critical theme – liars’ total details for the non-critical theme) = (liars’ total details for the critical theme – truth tellers’ total details for the critical theme). If we reformulate the terms of the equation, this corresponds to testing for the difference between the amount of detail reported by truth tellers for the critical theme and the amount of detail reported by liars for the non-critical theme. A *t*-test showed that the difference was not significant, *t*(66) = –1.116, *p* = 0.27. Therefore, the *t*-test supports the finding that the two confidence intervals (the one for the within-subjects measure for liars and the one for the between-subjects difference between truth tellers and liars for the critical theme only) do not overlap. However, in favor of the within-subjects measure, truth tellers showed only a small difference when talking about the two themes, Cohen’s *d* = 0.26 [–0.04, 0.57] and an investigator would probably not notice a difference (Cohen, 1988). In contrast, the effect size for liars was large, Cohen’s *d* = 1.63 [1.04, 2.21] and an investigator would arguably notice a difference (Cohen, 1988). Furthermore, there was no overlap between the confidence intervals of the two within-subjects measures.

## Discussion

In this experiment, we compared the efficacy of a within-subjects measure to that of a between-subjects measure to detect deception and tested the efficacy of a Theme-Selection approach to detect which part of the statement included a lie. Truth tellers reported the same amount of information about both themes, whereas liars reported less information for the theme they lied about than for the theme they told the truth about. Furthermore, larger differences between truth tellers and liars were found when focusing on within-subjects than on between-subjects comparisons focusing on the entire interview. This supports the idea that within-subjects measures are preferable to between-subjects measures.

The between-subjects comparisons, similarly to the within-subjects measure, also yielded strong effect sizes (especially when focusing on the critical theme only), yet this result has little applied value. To apply a between-subjects comparison an investigator should first determine a cut off score: what is the minimum amount of information that should be provided to consider a statement as truthful? This is an impossible task, for example, due to substantial individual differences between interviewees in how much information they volunteer in interviews (Nahari and Pazuelo, 2015; Vrij, 2016) and situational differences (some events are richer in detail than other events).

The fact that the effect size for the between-subjects comparison concerning the critical theme only was larger than that for the comparison accounting for the entire interview is due to the fact that liars’ statements concerning the critical theme only were entirely deceptive. Therefore, the advantage of our within-subjects measure over the between-subjects measure was reduced when examining the critical theme only. Yet, such a comparison is only possible when the statements have been split into the two themes.

Our results strengthen the idea that within-subjects measures are better than between-subjects measures, but they must be taken with caution as there are some limitations. For example, in the present experiment the deceptive part of the statement was entirely false^5^, which often would not mirror real life, as liars typically tell a mixture of truths and lies (Leins et al., 2013). Future research should explore the present approach when the false theme is itself a mixture a truth and lies.

In addition, the Theme-Selection approach also has a cut off score problem: Which difference in reported information between the two themes is required to decide that someone is lying? Although a within-subjects comparison controls for individual differences, the issue of situational differences is still relevant (some events are richer in detail than other events) and a difference in reporting details between the two events could appear also for truth tellers. The same applies when an interviewee has better memory for one theme than for the other theme. Hence, a difference in detail between themes does not automatically imply lying.

Finally, a liar may lie about both events in which case liars may report an equal amount of details for both themes. Therefore, a lack of difference does not automatically imply truth telling. It is therefore important that future research explores the effectiveness of the Theme-Selection Strategy when the two (or more) themes are intrinsically different and/or include a different amount of detail. We expect the approach to be less effective in those situations than in the current experiment. Future research could also explore in a lie detection experiment how the Theme-Selection strategy affects observers’ accuracy in discriminating between truth tellers and liars. In addition, in the present experiment we demarcated the two subthemes *a priori* and future research could explore how skilled interviewers are in separating subthemes in a story. Indeed, research is needed to explore if two (or more) interviewers split interviewees’ statement in the same way. For this to happen, a theme needs to be defined. In our view a theme is a cluster of pieces of information or events that are more related to each other than other pieces of information or events.

Furthermore, we did not apply any strategic questioning, but combining the Theme-Selection approach with strategic questioning may prove effective for separating truth tellers from liars and to understand what specific part(s) of the story is false. For example, the interviewer could ask unexpected questions (Lancaster et al., 2013; Vrij, 2018) for each theme and explore if the interviewee’s answers to such unexpected questions for one theme differ from those of another theme. Similarly, other measures such as ratios between verifiable and non-verifiable details (Nahari et al., 2014) or between complication and other types of details (Vrij et al., 2018) may be employed.

There was a methodological limitation in our study worth mentioning: We did not counterbalance the theme about which the interviewees lied. Although the rationale on which we built our experiment (different cognitive processes and strategies between truth telling and lying) is not affected by the theme about which the interviewees lies, the content of the various subthemes could have had an influence on the outcome. Therefore, counterbalancing should take place in future research. Lastly, our experiment was based on a role-playing situation. This has to be taken into account when considering the ecological validity of our results. A recent meta-analysis aiming to shed light on the issue concluded that “*[…]the findings from deception research are not laboratory artifacts- the detectability of deception remains stable across a variety of situational variables.*” (Hartwig and Bond, 2014, p. 667). Thus, although there are differences between real life and lab settings, lab research is still informative. In addition, we realize that there are difficulties in generalizing lab findings to real life when “stakes” play an important role in the lab study and interpretation of the lab research findings. However, in our experiment we focused on interviewees’ strategies. There is no reason to believe that truth tellers and liars in laboratory settings use different strategies to appear credible than truth tellers and liars in real life.

## Data Availability

The raw data supporting the conclusions of this manuscript will be made available by the authors, without undue reservation, to any qualified researcher.

## Ethics Statement

All procedures performed in studies involving human participants were in accordance with the ethical standards of the institutional and/or national research committee and with APA regulations.

## Author Contributions

NP conceived the idea for the study, designed the experiment, conducted the data analysis, interpreted the results, and wrote the manuscript. LC contributed to the design of the experiment, data analysis and interpretation of the results, writing up the manuscript, and provided feedback. AV contributed to the data analysis, the interpretation of the results, writing up the manuscript and provided feedback. All authors agreed on the final version of the manuscript.

## Conflict of Interest Statement

The authors declare that the research was conducted in the absence of any commercial or financial relationships that could be construed as a potential conflict of interest.

## Footnotes

1 Usually, any participant whose score is beyond the third quartile plus 1.5 times the interquartile range (IQR) or any participant whose score is below the first quartile minus 1.5 times the IQR is labeled as an outlier. However, the 1.5 by IQR formula has been criticized and a 2.2 multiplier is suggested instead (Hoaglin and Iglewicz, 1987). Therefore, to calculate our outliers we used the second formula.

2 Reliability coding on around 25% of the sample is usual practice in verbal lie detection research (Nahari and Vrij, 2015; Deeb et al., 2017). Although performing the ICCs only on a proportion of stimuli may have limitations, our high agreement shows that it was not problematic.

3 The fact that perceived credibility differed between veracity conditions further supports the assumption that the veracity manipulation worked. Indeed, liars believed to be less credible than truth tellers, which would be expected because of the illusion of transparency (Gilovich et al., 1998). We decided not to enter the *believability* score as a covariate because we asked how believable the participants thought they were *after* the interview was conducted and thought it to be unlikely that such score would influence the conclusion drawn from the analyses. Re-running the mixed model with the inclusion of *believability* covariate supported this assumption as the conclusions remained the same.

4 The Log(BF)s do not include the covariates as they are based on Bayesian Paired *t*-tests.

5 Twenty-one (65.6%) of lying participants did not report any true detail about the critical event, whereas the remaining eleven liars (35.4%) reported on average 12.6% of true details about the critical event.
